# Computed Tomography-Guided Diagnosis of Massive Cystojejunal Bleeding

**DOI:** 10.3390/diagnostics16193168

**Published:** 2026-09-29

**Authors:** Dragan Vasin, Goran Vukovic, Jelica Vukmirovic, Ksenija Mijovic, Aleksandar Pavlovic, Bojana Mladenovic, Danijela Sekulic, Aleksandra Pavic, Miona Jevtovic, Tijana Tomic

**Affiliations:** 1 Emergency Radiology Department, Center for Radiology, University Clinical Center of Serbia, Pasterova Str. No 2, 11000 Belgrade, Serbia; 2 Faculty of Medicine, University of Belgrade, Dr Subotica Str. No 8, 11000 Belgrade, Serbia; goranvukovic1965@gmail.com; 3 Clinic for Emergency Surgery, University Clinical Center of Serbia, Pasterova Str. No 2, 11000 Belgrade, Serbia

**Keywords:** cystojejunal anastomosis, computed tomography, surgery, bleeding

## Abstract

Acute gastrointestinal bleeding is a serious emergency condition which, without prompt and accurate diagnosis, can lead to severe hemorrhagic shock and death. The initial diagnosis relies on a clinical assessment of the bleeding site. If symptoms such as hematemesis and melena are present, an esophagogastroduodenoscopy (EGD) is typically the first diagnostic procedure. In cases of massive rectal bleeding, a computed tomography scan with angiography (CTA) is often used to identify the source and cause of the bleeding. Multiphasic CTA is the definitive first-line diagnostic standard, enabling prompt diagnosis and crucial pre-procedural planning for endovascular management in massive cystojejunal bleeding.

**Figure 1:**
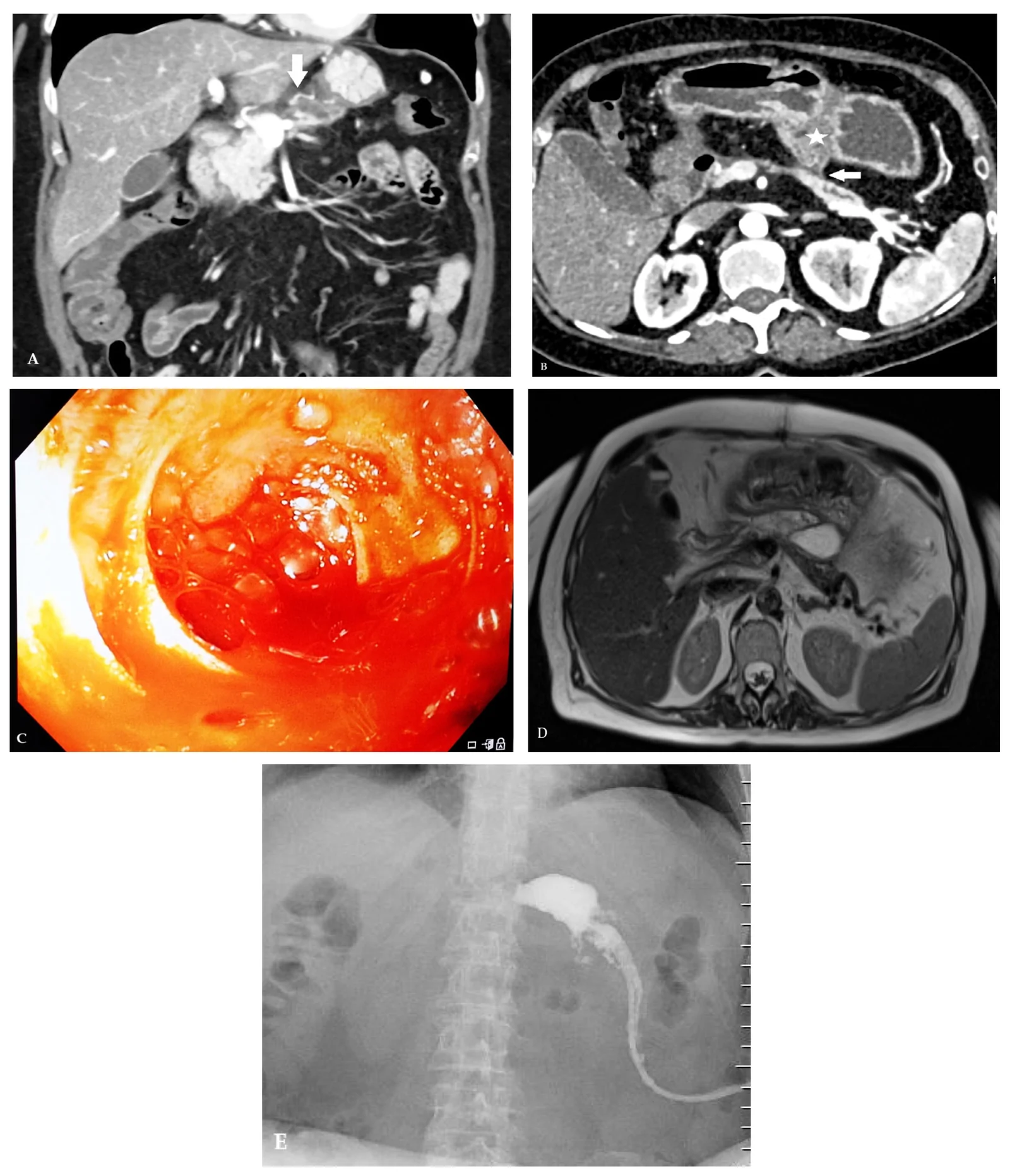
(**A**,**B**) Computed tomography angiography (CTA)—coronal and axial reconstruction showed irregular and wavy contours of juxta-anastomotic arterial blood vessel (arrow) in a 65-year-old woman who was admitted to the Emergency Center due to severe rectal bleeding. She had undergone surgery for a pancreatic pseudocyst two years previously, when CJA was performed, which is of critical importance for this patient. Upon admission, the patient was diagnosed with anemia (Hgb 80 g/L) and an abdominopelvic CTA was performed. The results showed no active bleeding and normal findings in the colon, as well as normal appearance of CJA. However, the juxta-anastomotic arterial blood vessel appeared to have irregular and wavy contours, and a “sentinel” hematoma was detected in juxta-anastomotic jejunal loop. Upper endoscopy was then performed, which did not reveal any signs of active bleeding or blood above Treitz’s ligament. In our patient, previous intestinal surgery was a potential site of bleeding, as shown by preoperative CTA. However, embolization was not possible for two reasons: CTA did not show active bleeding, and embolization of the artery at the pseudocystojejunostomy would result in ischemia and an uncontrolled pancreatic fistula. Despite receiving three blood transfusions, the patient’s hemoglobin levels dropped to 68 g/L and she required emergency surgery. During the surgery, a significant amount of blood was found in the small intestine, especially in juxta-anastomotic jejunal loop, as well as at the CJA. Star—cystojejuno anastomosis (CJA). An enterotomy was performed approximately 40 cm distal to the anastomosis, and intraoperative endoscopy revealed active bleeding from the small juxta-anastomotic artery, which could not be treated endoscopically (**C**). Deanastomosis was performed during the same operation, and the arterial blood vessel was sutured to achieve optimal hemostasis. The patient’s postoperative recovery was uneventful, with the only complication being a small pancreatic fistula and fluid collection (**D**), which was resolved through percutaneous drainage (**E**). Massive gastrointestinal hemorrhage secondary to internal drainage of a pancreatic pseudocyst via cystojejunostomy represents a rare but life-threatening post-interventional complication. A large number of cohort studies and some multicenter studies showed that CTA can diagnose acute gastrointestinal bleeding by determining the optimal bleeding area, suspicious bleeding artery, or intraluminal blood clots with high sensitivity and negative predictive value [1,2]. High pooled sensitivity and negative predictive value with an acceptable effective radiation dose were reported in meta-analyses. Secondly, although there is no optimal bleeding area, such as for the bleeding diverticulum in lower gastrointestinal bleeding, the possibility of finding the focus of bleeding was low when there was no evidence of hematoma in the CTA features [3,4]. CT angiography plays a pivotal role in rapidly detecting active extravasation distal to Treitz ligament and defining precise vascular anatomy to guide targeted transcatheter endovascular, endoscopic, or surgical interventions. When CT angiography fails to detect active contrast extravasation, indirect CT signs (sentinel clot, wall thickening, mesenteric fat stranding, and identifiable bowel pathology) may indicate the site of intermittent hemorrhage, which is essential for determining the appropriate therapeutic approach—as exemplified by the juxta-anastomotic arterial morphology in our case. The preferred treatment of acute upper gastrointestinal bleeding is usually endoscopic hemostasis, with interventional-radiological transcatheter embolization being used in a acute lower gastrointestinal bleeding. While emergency endoscopic evaluation was compromised by severe luminal clot pooling, CTA offered unobstructed spatial and vascular mapping of the peri-anastomotic territory [5]. If endoscopic findings are negative and there is no clear site of bleeding on CTA, surgery is the only option in cases of hemodynamic instability with active bleeding [6,7]. Multiphasic CTA is the definitive first-line diagnostic standard, enabling prompt diagnosis and crucial pre-procedural planning for management in massive cystojejunal bleeding [8,9,10]. Because active extravasation is often not captured, identification of the bleeding site relies heavily on subtle morphological alterations of the juxta-anastomotic arteries (e.g., pseudoaneurysms, caliber irregularities, focal wall disruption, or adjacent inflammatory encasement). It is our belief that the active role of radiologists in critical conditions, and their collaboration with the other critical figures of the emergency, will result in new technological improvements that will place CTA among the ever more successful variants of classical angiography.

## Data Availability

The original contributions presented in this study are included in the article/Supplementary Materials. Further inquiries can be directed to the corresponding author.

## Supplementary Materials

The following supporting information can be downloaded at: https://www.mdpi.com/article/10.3390/diagnostics16193168/s1.
